# Effects of anti-VEGF agents on rat retinal Müller glial cells

**Published:** 2010-05-01

**Authors:** Bin Guo, Yingli Wang, Yannian Hui, Xinguang Yang, Qinhua Fan

**Affiliations:** 1 Bayi hospital, Department of ophthalmology, Nanjing, China; 2 General Hospital of Coal Industry Ministry of China, Department of Ophthalmology Beijing, China; 3 Xijing Hospital, Fourth Military Medical University, Department of Ophthalmology, Xi'an, China; 4 No. 4 Hospital of Xi'an, Department of Ophthalmology, Xi'an, China

## Abstract

**Purpose:**

To evaluate the effects of an anti-rat vascular endothelial growth factor antibody (ARVA) and bevacizumab (Avastin) on rat retinal Müller glial cells (RMGCs) in vivo and in vitro.

**Methods:**

Rat RMGCs were identified and cultivated, and were then treated with bevacizumab (0.1, 0.25, and 1 mg/ml), ARVA (0.1, 0.5, and 1 µg/ml), or 1 mg/ml of rat immunoglobulin G (IgG) for 12, 24, 48, and 72 h. The numbers of viable RMGCs were determined using a trypan blue dye exclusion assay and a methyl thiazolyl tetrazolium colorimetric assay. In the in vivo study, the rats received intravitreal injections of 5 µl bevacizumab (3.75 mg/ml), ARVA (15 µg/ml), and rat IgG (1 mg/ml). The electroretinogram was recorded. Seven days after the injections, histopathologic changes and glial fibrillary acidic protein expression of RMGCs in the retina were analyzed by immunohistochemistry with hematoxylin-eosin and fluorescent staining.

**Results:**

After exposure to bevacizumab at various concentrations for various periods of time, the stained cell numbers and optical density values of mitochondrial dehydrogenase activity of RMGCs had no significant differences (p>0.05) from those of the control group and IgG medium. In the stained cells, ARVA demonstrated a dose-dependent increase. Compared with those treated for 12 and 24 h, the increase of stained cells treated with 0.5 and 1 µg/ml ARVA at 48 and 72 h was very significant (p<0.01). The optical densities of RMGCs exposed to 0.5 and 1 µg/ml of ARVA at 48 and 72 h were significantly lower than cells exposed to a fresh culture medium (p<0.01). The histology of both treated and control eyes after intravitreal injection was similar and showed no anatomic signs of toxicity. There were no obvious glial fibrillary acidic protein upregulations of RMGCs in all groups. The scotopic electroretinogram responses to flashes of light in the control and treated eyes had similar b-wave amplitudes.

**Conclusions:**

Intravitreal bevacizumab and ARVA had no short-term, direct retinal toxicity in rats. Bevacizumab exerts no inhibition on rat RMGCs, while ARVA at higher doses (over 0.5 µg/ml) may be harmful to the growth of RMGCs.

## Introduction

Neovascular eye diseases including diabetic retinopathy (DR) and age-related macular degeneration (AMD) have become leading causes for substantial and irreversible vision loss among the populations of industrialized nations [1-3]. Numerous studies have demonstrated that vascular endothelial growth factor (VEGF) is a key cytokine responsible for the formation of retinal angiogenesis in DR and choroidal neovascularization (CNV) in AMD [2,4-6]. Recent advances in understanding the pathogenesis leads to novel efficacious pharmaceutical treatment of neovascular diseases, which aims at specific aspects of angiogenesis and anti-VEGF agents [2,7]; Pegaptanib and Ranibizumab were approved for treating neovascular AMD by the Food and Drug Administration and the European Medicines Agency [8]. Bevacizumab is a full length recombinant, humanized antibody with a molecular weight of 149-kDa, binding to all isoforms of VEGF and interfering with its binding to receptors, thus inhibiting its signal pathway [9]. Although bevacizumab is not formally approved for neovascular eye disease treatment, a few multi-center studies have demonstrated that intravitreal injections of bevacizumab have a beneficial effect on neovascular AMD and DR, improving visual acuity and reducing retinal edema [10,11].

Previous studies have evaluated the safety of intravitreal anti-VEGF agent injections in patients and animals using physiologic testing and histopathologic analysis [12,13]. Some groups reported that bevacizumab showed no retinal toxicity in rats [14,15]. However, Fuh et al. pointed out that bevacizumab is human-specific and does not react with rat VEGF because of an amino acid substitution in the bevacizumab-binding site [7,16]. Therefore, the safety of anti-rat VEGF antibody (ARVA) should be tested on rat retinas.

The retinal Müller glial cell (RMGC) is the principal glial cell of the vertebrate retina. Retinal astrocytes are in contact with the superficial vascular plexus via processes wrapped around the vessels and ensheath all retinal neuronal somata [4]. Our previous study showed expression of VEGF and VEGF receptors (Flk-1, Flt-1) in rat RMGCs. Partial neuroprotective effects by exogenous VEGF on RMGCs were also observed [5]. As intravitreal bevacizumab has increasingly been used to treat neovascular eye diseases, its safety with RMGC or glial cells should be further studied.

This study is the first of its kind on the in vitro effects of bevacizumab and anti-rat VEGF agent on RMGCs, as well as on the in vivo effects of the intravitreal administration of the two agents on glial cells.

## Methods

### Animals

Wistar rats (6 weeks and 3–5 days old) were purchased from Laboratory Animal Supply Center, Fourth Military Medical University, China. The animals were kept under standard laboratory conditions with a 12 h:12 h light-dark cycle and were supplied with adequate food and water. All experiments were conducted in accordance with the Animal Care and Use Committee and the ARVO Statement for the Use of Animals in Ophthalmic and Vision Research.

### Isolation, culture, and identification of rat retinal Müller glial cells

Rat RMGCs were isolated according to the methods described in a previous report [6]. Briefly, the enucleated eyes of Wistar rats were placed in Dulbecco’s Modified Eagle Medium (DMEM, Sigma-Aldrich, St. Louis, MO) containing 100 µg/ml streptomycin and 100 U/ml penicillin at room temperature for 30 min. Twenty retinas were minced and the suspension filtered through a 53 µm nylon mesh. Then, the filtrate was collected and centrifuged at 500× g for 10 min and the suspension were seeded onto clean two 75 cm2 tissue culture flasks at 37 °C in a humidified 5% CO_2_ atmosphere. The medium was changed and non-attached cells were discarded 24 h later. Retinal Müller glial cells were grown in DMEM containing 200 ml/l heat-inactivated FBS, 2 mM L-glutamine, 2 mM sodium pyruvate, 20 mM 4-(2-hydroxyethyl)-1-piperazineethanesulfonic acid (HEPES), 10 mg/ml nonessential amino acids, 100 µg/ml streptomycin, and 100 U/ml penicillin. The above medium was left unchanged for 5 to 6 days and then was replaced every 3 to 4 days. Cells were dissociated from the flask surface with 2.5 mg/ml trypsin and 0.2 mg/ml EDTA (EDTA; TE) by incubation at 37 °C. Cells of passages 2–4 were used for the experiments. Müller glial cells were immunostained with anti-vimentin monoclonal antibody (V9 clone, 1:50 dilution, Dako, Copenhagen, Denmark) and rabbit anti-glial fibrillary acidic protein (GFAP) polyclonal antibody (1:50 dilution; Dako). The second antibody was fluorescein isothiocyanate (FITC) labeled immunoglobulin (1:200 dilution; Dako).

### Cells treated with anti-vascular endothelial growth factor agents

Cells were cultured to 70% confluence, and cell cultures were adapted into fresh culture medium 12 h before the addition of anti-VEGF agents, which was serially diluted in culture medium to appropriate concentrations just before use. 0.1, 0.25, and 1 mg/ml bevacizumab (Genentech/Roche, San Francisco, CA) and 0.1, 0.5, and 1 µg/ml of an anti-rat VEGF monoclonal antibody (Clone 123704; ARVA; Leinco Technologies, Inc. St. Louis, MO) were added to the RMGCs. The ARVA could block rat VEGF164—similar to bevacizumab’s specific neutralizing human VEGF165—but not all isoforms. As controls, RMGCs were treated with fresh culture medium with and without 1 mg /ml of rat immunoglobulin G (IgG), a nonspecific antibody. In our preliminary study, specificity of bevacizumab and ARVA to rat RMGCs was tested with western blotting. Bevacizumab could not bind any VEGF isoforms while ARVA could specifically neutralize VEGF-164 expressed by rat RMGCs. The amount of ARVA used in a previous report [7] was pretested. After treated by ARVA with various concentrations (0.1, 1, and 10 µg/ml), VEGF expression in the cultural medium and the cytoplasm by RMGCs were measured using an enzyme linked immunosorbent assay and an immunofluorescence method. VEGF could not be detected after treated by ARVA at 1 and 10 µg/ml concentrations, but it could be detected after treated by 0.1 µg/ml of ARVA. Furthermore, ARVA at a concentration of 0.5 µg/ml was added in vitro study. Treated or untreated cells were collected at 12, 24, 48, and 72 h for cell viability analysis. Cell viability was then assessed using two methods: viable cells were directly counted with trypan blue for the exclusion of dead cells and mitochondrial dehydrogenase activity was quantified using a methyl thiazolyl tetrazolium (MTT) colorimetric assay. Each measurement was performed at least three times.

### Intravitreal injection

The rats were anesthetized intraperitoneally with ketamine (70 mg/kg, Sigma) and xylazine (10 mg/kg, Sigma) and pupils were dilated with Mydrin-P eye drops (Santen Pharmaceutical Co., Ltd., Osaka, Japan). Five µl of bevacizumab (3.75 mg/ml), or ARVA (15 µg/ml), or rat IgG (1 mg/ml) was then injected into the vitreous cavity of the right eye of each rat (n=6), as previously described [17]. The intravitreal injection was performed with a 30-gauge needle attached to a syringe through the sclera 1 mm posterior to the limbus, guided by a stereoscopic microscope, with care taken to avoid lens and retinal injury. Five µl of physiologic saline was injected intravitreally in the left eye as control.

### Electroretinogram

Before the intravitreal injection and 7 days after injection, the Wistar rats were dark-adapted overnight, anesthetized, and their pupils were dilated. Electroretinography (ERG) was performed using the GT-2000 system (Guo-Te medical system, Chongqing, China). The scotopic flash responses were recorded from the treated and control eyes, using self-made silver chloride corneal electrode rings. The reference and ground electrodes were made of stainless steel surgical needles, and were placed subcutaneously under each eye and in the tail. Light stimulus was presented using a computer-controlled miniature Ganzfeld flash unit in a series of nine flashes with a maximum intensity of 2.0 cd•s•m^−2^, and responses were recorded. Scotopic a-wave amplitudes were measured from baseline to the corneal negative peak and b-wave amplitudes measured from the corneal negative peak to the major corneal positive peak after subtracting any contributions due to oscillatory potentials. Three responses were then averaged for each flash intensity.

### Histologic examination

Following deep anesthesia, all rats were perfused first with Ringer’s solution followed by 2% paraformaldehyde in 0.1 M phosphate buffered saline (PBS) at pH 7.4. The eyeballs were then removed and postfixed in the same fixative for 4 h before being transferred into PBS containing 200 mg/ml sucrose and kept overnight at 4 °C. Two eyes in each group were dehydrated in a series of graded alcohols, and embedded in paraffin. Sections of 5 µm thickness were obtained using a microtome, and were stained with hematoxylin-eosin. Slides were examined under a light microscope. The other four eyes in each group were used for immunohistologic analysis of GFAP. Frozen sagittal sections of the eyes were cut at 20 µm thickness and mounted on gelatin-coated slides. Mounted sections were washed for 20 min in PBS at pH 7.4. They were then incubated overnight with the GFAP monoclonal antibody (1:50 dilution; Dako). These antibodies were diluted with PBS containing 0.1% Triton X-100. After incubation, sections were rinsed in PBS for 15 min and reacted using the Vectastain ABC Kit (PK4002, Vector Laboratories, Burlingame, CA) against mouse IgG for 1 h. They were then treated with 3,3′-diaminobenzidine tetrahydrochloride as the peroxidase substrate. After this, the sections were counterstained with 1% methyl green, dehydrated, and mounted in Permount. Finally, the fluorescent sections were observed and photographed with a confocal laser scanning microscope (CLSM FV-300; Olympus, Tokyo, Japan).

### Statistical analysis

Statistical analysis was performed with SPSS 13.0 (SPSS, Chicago, IL). The cell viability differences among the various treatment groups (treated with bevacizumab at various concentrations) and rat IgG for various periods were evaluated by ANOVA and the Student *t*-test. The Mann–Whitney test and Wilcoxon matched-pair signed rank test were used to evaluate b-wave amplitude differences between control and treated eyes before and 7 days after injections. A p<0.05 was considered statistically significant.

## Results

### Effect of bevacizumab and ARVA on rat retinal Müller glial cells viability in vitro

**Figure 1:**
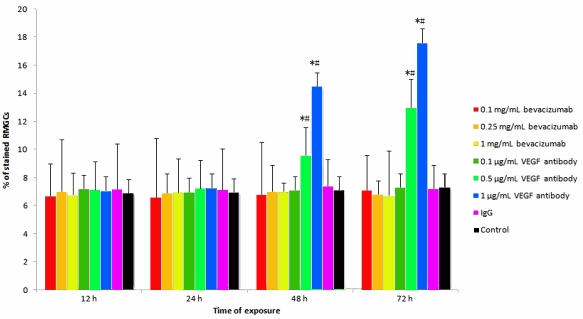
Trypan blue exclusion assay showing the percentages of trypan blue-positive rat retinal Müller glial cells (RMGCs) after exposure to bevacizumab (0.1, 0.25, and 1 mg/ml), anti-rat vascular endothelial growth factor (VEGF) antibody (0.1, 0.5 and 1 μg/ml), 1 mg/ml of rat immunoglobulin G (IgG), and control of fresh medium for 12, 24, 48, and 72 h . Data were shown as mean±SD (n=5). The percentage of stained RMGCs at 48 and 72 h exposures to anti-rat VEGF antibody at the concentrations of 0.5 and 1 μg/ml increased remarkably. The percentage of stained RMGCs was not affected after exposure to bevacizumab, IgG, and fresh control medium. The experiment was performed at least three times with similar results. *, p<0.01 significantly differs from control. #, p<0.01 represents significant difference between 48 and 72 h in 0.5 or 1 µg/ml ARVA group.

As shown in Figure 1, cultures of cells exposed to bevacizumab at various concentrations and rat IgG for various periods showed similar percentages of stained cells with trypan blue. In contrast, RMGCs exposed to anti-rat VEGF antibody for 48 or 72 h demonstrated a dose-dependent increase in the percentage of trypan blue-stained cells; the difference in the percentage of stained cells between 48 and 72 h was particularly remarkable after exposure to anti-rat VEGF antibody at the concentrations of 0.5 and 1 µg/ml.

**Figure 2:**
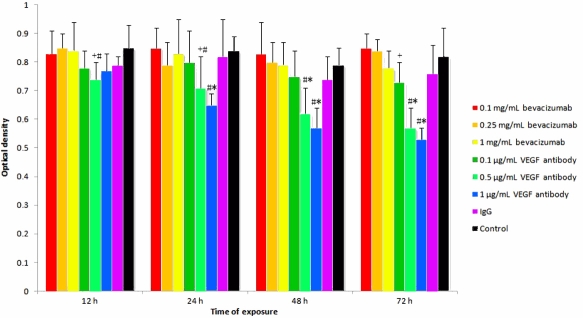
Methyl thiazolyl tetrazolium (MTT) assay showing optical densities of rat retinal Müller glial cells (RMGCs) after exposure for 12, 24, 48, and 72 h to bevacizumab (0.1, 0.25, and 1 mg/ml), anti-rat vascular endothelial growth factor (VEGF) antibody (0.1, 0.5 and 1 μg/ml), 1 mg/ml rat immunoglobulin G (IgG), and fresh medium as a control. Data were shown as the mean±SD (n=5). The optical densities of RMGCs exposed for 48 and 72 h to anti-rat VEGF antibody at the concentrations of 0.5 μg/ml and 1 μg/ml decreased remarkably. The optical densities of RMGCs were not affected after exposition to bevacizumab, IgG, and fresh control medium. Each value represents the mean of three replicates. *, p<0.01 and +, p<0.05 differs from the control. #, p<0.01 represents significant difference between various exposure time in 0.5 or 1 µg/ml ARVA group.

A MTT colorimetric assay was used to quantify mitochondrial dehydrogenase activity. As shown in Figure 2, 12 or 24 h after exposure, there was no significant difference in optical density (an indicator of mitochondrial function) among the groups. However, at 48 and 72 h after exposure to anti-rat VEGF antibody, the cultures of RMGCs had significantly lower optical densities than the cells exposed to fresh culture medium. The RMGCs exposed to bevacizumab at various concentrations or exposed to rat IgG and fresh culture medium for various periods showed similar optical densities.

### Histologic analysis

**Figure 3:**
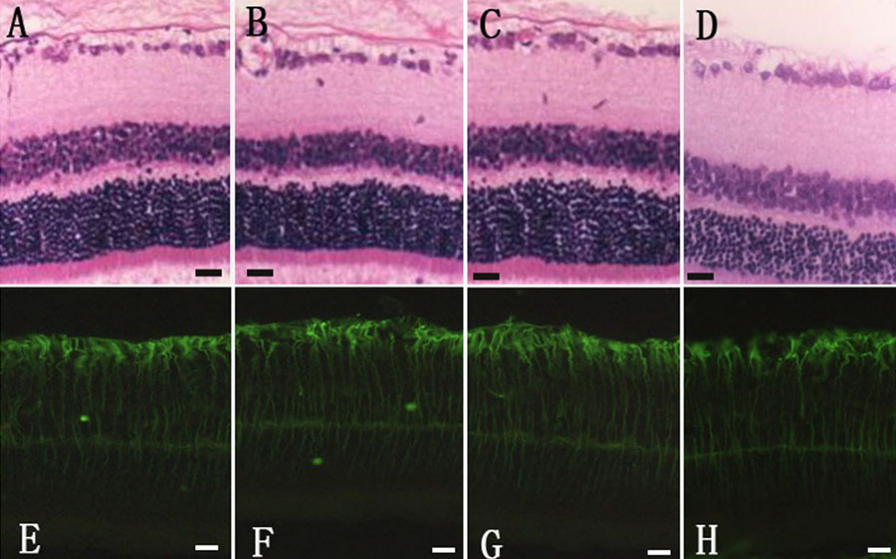
The histologic sections of the retina with hematoxylin and eosin (HE) staining 7 days after intravitreal injection of 5 µl of 3.75 mg/ml bevacizumab (**A**), 15 µg/ml anti-rat vascular endothelial growth factor (VEGF) antibody (**B**), 1 mg/ml rat immunoglobulin G (IgG; **C**), and physiologic saline (**D**). **E**–**H** are the fluorescence images of glial fibrillary acidic protein (GFAP)-immunolabeled sections 7 days after intravitreal injection of bevacizumab (**E**), anti-rat VEGF antibody (**F**), rat IgG (**G**), and physiologic saline (**H**). The scale bar represents 20 µm.

Light and confocal laser scanning microscopy was performed in all eyes. The histology of eyes in both the treated and control group after intravitreal injection was not distinguishable and no anatomic sign of toxicity was observed. There was no obvious upregulation of glial fibrillary acidic protein (GFAP) in the RMGCs of any group (Figure 3).

### Effect of intravitreal injection of anti-vascular endothelial growth factor agents on electroretinogram

**Figure 4:**
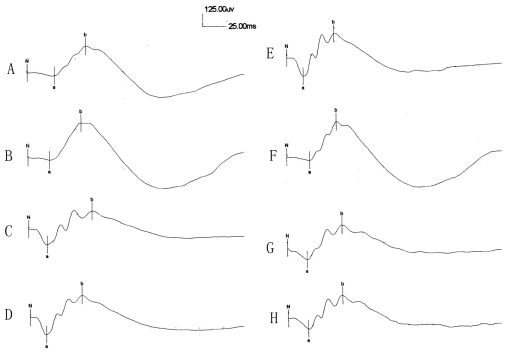
Example of scotopic electroretinogram (ERG) in groups before bevacizumab (**A**), anti-rat vascular endothelial growth factor (VEGF) antibody (**B**), rat immunoglobulin G (IgG; **C**), and physiologic saline (**D**) injection, and 7 days after intravitreal injection of 5 µl of 3.75 mg/ml bevacizumab (**E**), 15 µg/ml anti-rat VEGF antibody (**F**), 1 mg/ml rat IgG (G), or physiologic saline (**H**). No significant difference in b-wave amplitude was observed between bevacizumab, anti-rat VEGF antibody, rat IgG, and physiologic saline injection groups.

The effects of anti-VEGF agent intravitreal injections on retinal function were assessed by ERG in this study as described previously [18]. Electroretinogram analysis was based on measurements of the b-wave amplitude from the trough of the a-wave to the peak of the b-wave. The “Müller cell hypothesis” suggests that the ERG b-wave comes from changes in RMGC membrane potential, which arise from light-induced fluctuations of extracellular potassium concentration due to depolarizing retinal neurons [19,20]. Scotopic ERG responses to flash light were evaluated in dark-adapted rats before and 7 days after intravitreal injection (Figure 4). The b-wave amplitude of the bevacizumab group, anti-rat VEGF antibody group, rat IgG group, and physiologic saline group were 320.1±34.4, 335.3±19.7, 324.3±13.1, and 344.2±30.7 uv before injections, respectively. Seven days after intravitreal injections, the values were 297.7±22.1, 321.6±35.7, 314.3±47.3, and 329.3±36.4 uv, respectively. Intravitreal injection of anti-VEGF agents was not observed to decrease b-wave amplitude significantly in any group.

## Discussion

In the past few years, dramatic new therapies with anti-VEGF antibody have been developed as treatment options for patients having neovascular eye diseases, especially AMD [2,3]. The safety and efficacy of anti-VEGF agents in the treatment of these diseases has been evaluated and the results indicate that the intravitreal administration of anti-VEGF agents has limitations [21-23]. To date, many published data showed that the bevacizumab agent was not harmful to retina in vivo or to various retinal cells in vitro, while Spitzer et al. reported that bevacizumab could exert a moderate growth inhibition on pig choroidal endothelial cells and that high doses of bevacizumab may be harmful to ARPE-19 cells, a human retinal pigment epithelial cell line, in vitro [14]. Klettner et al. found that bevacizumab moderately impairs the proliferation of porcine RPE cells and reduces their phagocytic function [24,25]. It has also been found that anti-VEGF agent has minimal effects on the permeability or selectivity of RPE tight junctions [26]. Retinal Müller glial cells are a critical component in the retinal-blood barrier. With anti-VEGF agent clinical application in neovascular eye diseases, features of RMGC might be influenced. Currently, there are no reports on the in vitro or in vivo toxic effects of bevacizumab on RMGCs.

Retinal Müller glial cells span the entire thickness of the inner retina, secret proangiogenic, and antiangiogenic factors, such as VEGF, and may act as modulators of immune and inflammatory responses under pathological conditions [27-29]. Since intravitreal bevacizumab penetrates the retina from the vitreal side, VEGF in RMGCs were inevitably responded to anti-VEGF agents. The aim of this study was to examine the effect of bevacizumab on RMGCs, which may interfere with anti-VEGF agent treatment.

Retinal Müller glial cell gliosis is a response to virtually every pathological alteration of the retina [30]. Gliotic RMGC releases factors such as VEGF, which may have both neuroprotective and detrimental effects as VEGF may exacerbate neovascular disease progression [31]. The most sensitive non-specific response to retinal diseases and injuries, which can be used as a universal early cellular marker for retinal injury, is the upregulation of glial fibrillary acidic protein (GFAP) [32,33].

In the present study, no GFAP upregulation of RMGCs was observed in the retina in vivo after intravitreal bevacizumab injection at a high concentration of 3.75 mg/ml. No evidence for retinal toxicity resulting from the intravitreal bevacizumab injection in rat model were found based on electrophysiology in this study. In addition, the effect of bevacizumab on cultured RMGCs was also examined. No toxic or anti-proliferative effect on RMGCs was observed with 0.1 to 1 mg/ml bevacizumab.

However, since bevacizumab is human-specific and does not react with rat VEGF, anti-rat VEGF antibody was further examined in vivo and in vitro on rat RMGCs with similar assays as used by Iriyama et al. [7].

When 0.5 and 1 µg/ml anti-rat VEGF antibody was used, an increased percentage of cell death and anti-proliferation effect on RMGCs were observed after 48 and 72 h incubation. Previous studies found that VEGF exerts neuroprotective effects on various cultured neurons and glial cells [17] and RMGCs might lose these neuroprotective effects after RMGCs secreted VEGF consumed by anti-rat VEGF antibody. It has been reported that VEGF, one of the hypoxia inducible factor-1 target gene products, shows protective effects against neuron apoptosis induced by brain ischemia [34]. Hypoxia inducible factor-1, possibly implicated in pyruvate kinase induction, has regulating effects on glial cells death in hypoxic environment [35]. Therefore, in in vitro study, ARVA blocked VEGF and might induce apoptosis of RMGCs. However, intravitreal injected anti-rat VEGF antibody neither increased the expression of GFAP in the retina nor decreased the amplitude of b-wave in rat ERG, suggesting that VEGF antibody is not associated with significant retinal toxicity. Although the expression of GFAP in RMGCs was not been observed after intravitreal injection, the direct effects of anti-VEGF agent on RMGCs cannot be excluded.

In summary, bevacizumab and anti-rat VEGF agent did not appear to be toxic to the retina in rats. This study supports the safety of intravitreal anti-VEGF agent treatments of choroidal neovascularization and retinal neovascular diseases. Further studies are still required to evaluate the long-term safety of the drugs.
